# Oligogenic inheritance in severe adult obesity

**DOI:** 10.1038/s41366-024-01476-9

**Published:** 2024-01-31

**Authors:** Sumaya Almansoori, Suzanne I. Alsters, Andrianos M. Yiorkas, Nikman Adli Nor Hashim, Robin G. Walters, Harvinder S. Chahal, Sanjay Purkayastha, Nader Lessan, Alexandra I. F. Blakemore

**Affiliations:** 1 Department of Metabolism, Digestion and Reproduction, Imperial College London, London, UK; 2 Department of Life Sciences, College of Health, Medicine and Life Sciences, Brunel University London, London, UK; 3 College of Medicine, Mohammed Bin Rashid University of Medicine and Health Sciences, Dubai Healthcare City, Dubai, United Arab Emirates; 4 Genome Center, Department of Forensic Science and Criminology, Dubai Police GHQ, Dubai, United Arab Emirates; 5 South West Thames Regional Genetics Service, St George’s University Hospitals NHS Foundation Trust, London, UK; 6 Institute of Biological Sciences, Faculty of Science, Universiti Malaya, Kuala Lumpur, 50603 Malaysia; 7 Centre for Drug Research in Systems Biology, Structural Bioinformatics and Human Digital Imaging (CRYSTAL), Universiti Malaya, Kuala Lumpur, 50603 Malaysia; 8 Nuffield Department of Population Health, University of Oxford, Oxford, UK; 9 MRC Population Health Research Unit, University of Oxford, Oxford, UK; 10 Imperial Weight Centre, Imperial College Healthcare NHS Trust, St Mary’s Hospital, Praed Street, London, W2 1NY UK; 11 Section of Investigative Medicine, Division of Diabetes, Endocrinology and Metabolism, Imperial College London, Hammersmith Campus, Hammersmith Hospital, 6th Floor Commonwealth Building, Du Cane Road, London, W12 0NN UK; 12 Department of Surgery and Cancer, Imperial College London, London, UK; 13 Imperial College London Diabetes Centre Abu Dhabi, Abu Dhabi, United Arab Emirates; 14 College of Medicine, Nursing, and Health Science, University of Galway, Galway, Republic of Ireland

**Keywords:** Genetics, Obesity

## Abstract

**Background/objective:**

The genetic architecture of extreme non-syndromic obesity in adults remains to be elucidated. A range of genes are known to cause monogenic obesity, but even when pathogenic mutations are present, there may be variable penetrance.

**Methods:**

Whole-exome sequencing (WES) was carried out on a 15-year-old male proband of Pakistani ancestry who had severe obesity. This was followed by family segregation analysis, using Sanger sequencing. We also undertook re-analysis of WES data from 91 unrelated adults with severe obesity (86% white European ancestry) from the Personalised Medicine for Morbid Obesity (PMMO) cohort, recruited from the UK National Health Service.

**Results:**

We identified an oligogenic mode of inheritance of obesity in the proband’s family—this provided the impetus to reanalyze existing sequence data in a separate dataset. Analysis of PMMO participant data revealed two further patients who carried more than one rare, predicted-deleterious mutation in a known monogenic obesity gene. In all three cases, the genes involved had known autosomal dominant inheritance, with incomplete penetrance.

**Conclusion:**

Oligogenic inheritance may explain some of the variable penetrance in Mendelian forms of obesity. We caution clinicians and researchers to avoid confining sequence analysis to individual genes and, in particular, not to stop looking when the first potentially-causative mutation is found.

## Introduction

Obesity is a genetically heterogeneous disorder that is known to occur in multifactorial, monogenic, or syndromic forms. The increasing availability of whole-exome sequencing (sequencing of the genomic regions encoding proteins and closely linked regulatory regions), has improved our understanding of those sub-forms of obesity that are inherited in a simple Mendelian fashion. However, we are still some way from appreciating the full complexity of its genetic architecture, including explanations for the variable penetrance of known pathogenic mutations (whether or not a carrier of a harmful mutation expresses the expected trait) [1, 2]. It is possible that, at least in some cases, more than one genetic deficit may be required for production of a severe phenotype. The concept of inheritance patterns more complex than simple Mendelian dominance or recessivity has been suggested previously in obesity syndromes and other conditions, but the implications of this in people ascertained only for severe obesity remains unclear [3–8].

Here, we present results suggesting oligogenic inheritance of obesity (whereby a small number of mutations act together to cause the phenotype), first in a family trio where the proband has obesity and learning difficulties, and subsequently in unrelated adults with severe obesity, but without known learning disability or dysmorphism.

## Results

### Family segregation analysis: proband 1

**Fig. 1:**
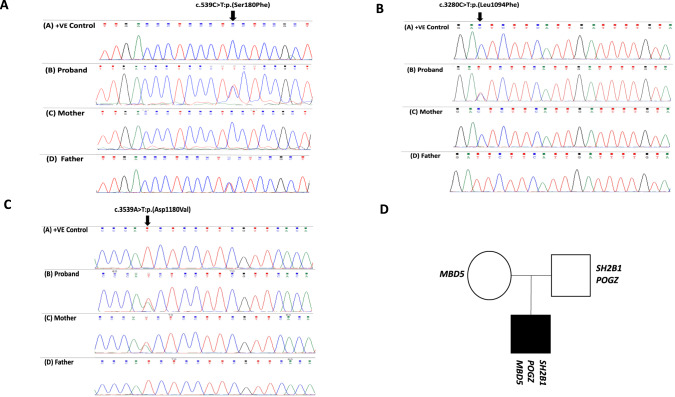
Sanger Sequencing Chromatograms and Family Pedigree of Proband 1. **A** Chromatograms of Sanger sequencing demonstrating heterozygosity for SH2B1:c.539 C > T in the proband and his healthy parents. **B** Chromatogram of Sanger sequencing demonstrating heterozygosity for POGZ:c.3280 C > T in the proband and his healthy father. **C** Chromatogram of Sanger sequencing demonstrating heterozygosity for MBD5:c.3539 A > T in the proband and his healthy mother. **D** Pedigree of the family and the putatively deleterious variants in each individual.

Proband 1, a 22-year-old male of South Asian origin with obesity, attention deficit hyperactivity disorder (ADHD), depression, and sleep apnoea, underwent whole-exome sequencing (WES) for diagnostic purposes. A heterozygous deleterious mutation in the sarcoma (Src) homology 2 B adaptor protein 1 (*SH2B1*) gene c.539 C > T:p.(Ser180Phe), with a CADD score of 23.6 (Table 1 and Fig. 1A) was detected in initial analysis. The identified variant is located close to the site of a variant (A175N) previously reported in individuals with severe early-onset obesity which was shown to disrupt the function of NGF-induced neuronal differentiation. Subsequent characterisation of the proband’s parents by Sanger sequencing revealed that the father without obesity was carrying the same mutation (Fig. 1B), raising questions about penetrance or pathogenicity of this variant. Re-evaluation of the original exome sequence data revealed two further deleterious variants, that had previously been overlooked on the assumption that the *SH2B1* mutation was causative for the phenotype: c.3539 A > T:p.(Asp1180Val) in *MBD5*, and c.3280 C > T:p.(Leu1094Phe) in *POGZ*, with CADD scores of 22.9 and 24.2 respectively (Fig. 1B, C). All mutations were rare with ≤0.1% minor allele frequency (MAF) in gnomAD, as shown in Table 1. Considered individually, none of the three variants showed co-segregation with the phenotype, but the proband had inherited all three.

**Table 1:** Details of the putatively deleterious variants identified in proband 1.

Gene	MOI	Variant type	Variant	RS ID	MAF (gnomAD all)	MAF (gnomAD**)	CADD	Zygosity
SH2B1	AD	Missense	NM_001145795: c.539 C > T:p.(Ser180Phe)	rs144107554	0.00002131	0.00003270	23.6	Het
POGZ	AD	Missense	NM_001194937: c.3280 C > T:p.(Leu1094Phe)	rs768284272	0.0001591	0.001241	24.2	Het
MBD5	AD	Missense	NM_018328: c.3539 A > T:p.(Asp1180Val)	rs752035001	0.00007163	0.0004246	22.9	Het

*MOI* Mode of Inheritance, *AD* Autosomal dominant, *MAF (gnomAD all)* Minor allele frequency for all populations combined in gnomAD database version v2.1.1, MAF (gnomAD**) Population-specific minor allele frequency from gnomAD database v2.1.1 based on the subject’s self-reported ancestry, *CADD* Combined Annotation Dependent Depletion.

### Oligogenic obesity in severe adult obesity without learning disability or dysmorphism

After identifying the initial case with potentially oligogenic mode of inheritance, re-analysis was performed of WES data from 91 unrelated adults (86% white European ancestry) with severe obesity from the PMMO cohort, recruited from UK bariatric services [9]. In this analysis, two further cases of apparent oligogenic inheritance of obesity were identified. The identified oligogenic events affected pairs of genes *SH2B1*/*RAI1* and *SETD2*/*POGZ*, as summarised in Table 2. Each variant was a missense variant in heterozygous state (which matched the known autosomal dominant mode of inheritance) and had a CADD score greater than 20. Although these variants affect evolutionarily-conserved residues; this is the first report that they are potentially causative of obesity. Notably, for each of these pairs, one of the affected genes was among those mutated in proband 1.

**Table 2:** Summary of cases with oligogenic inheritance of severe obesity identified from re-analysis of existing WES data.

Case	Ancestry	Gender	Gene	MOI	Rs ID/Pos	Type of variant	Variant	CADD	MAF (gnomAD all)	MAF (gnomAD**)	
2	White British	F	*SH2B1*	AD	rs772678200	Missense	c. 1633G > A:p.(Gly545Ser)	29	0.0000244	0.00004765	North-western European
			*RAI1*	*AD*	rs113208290	Missense	c. 1142 C > T:p.(Ala381Val)	24	0.003643	0.004670	North-western European
3	White British	F	*POGZ*	AD	1:151381292	Missense	c.T1912>C:p.(Tyr638His)	28.9	–	–	–
			*STED2*	AD	rs780019200	Missense	c.6703 G > C;p.(Val2235Leu)	23.4	0.00001769	0.000008800	North-western European

*MOI* Mode of Inheritance, *AD* Autosomal dominant, *MAF (gnomAD all)* Minor allele frequency for all populations combined in gnomAD database version v2.1.1, MAF (gnomAD**) Population-specific minor allele frequency from gnomAD database v2.1.1 based on the subject’s self-reported ancestry, *CADD* Combined Annotation Dependent Depletion.

## Discussion

To our knowledge, this is the first report of apparent oligogenic inheritance of severe (non-syndromic) obesity, and is potentially of clinical importance in explaining the observed variable penetrance of known pathogenic mutations.

The initial finding of apparent oligogenic inheritance was in a proband (proband 1) with obesity and learning difficulties. Exome sequencing revealed heterozygous predicted-deleterious variants in three separate obesity-relevant genes: *SH2B1*, *POGZ* and *MBD5*. However, each gene is reportedly autosomal dominant, and one of these variants was present in the healthy mother and two in the healthy father. This suggests that an oligogenic inheritance model might be responsible for the phenotype of the proband.

The first identified variant in proband 1 was in a known obesity gene, *SH2B1*. The variant is located close to a variant (p.A175N) previously reported in individuals with severe early-onset obesity, which was shown to disrupt the function of NGF-induced neuronal differentiation [10]. Mutations in the *POGZ* gene cause White-Sutton syndrome, which has a highly variable phenotype including obesity, developmental delay, language and speech delay, motor delay, microcephaly, and non-specific vision problems [11–13]. *POGZ* encodes a zinc finger protein believed to have an important role in mitotic progression and possibly in neuronal proliferation [14, 15]. The third predicted-deleterious variant of interest in this proband is in *MBD5*, which encodes a member of the MBD family (which also includes the *MECP2* gene, a causal gene for Rett syndrome) [16]. The encoded protein is highly expressed in brain, oocytes and testis and is thought to have a role in heterochromatin and epigenetic reprogramming [16]. The *MBD5* gene has been identified as a potential causative locus contributing to 2q23.1 microdeletion syndrome: The phenotypic effects of genomic disruption of *MBD5* (ranging from point mutation to deletion or duplication) have considerable overlap with the clinical phenotype of 2q23.1 deletion syndrome, and have been termed *MBD5*-associated neurodevelopmental disorder (MAND) [17, 18]. Haploinsufficiency of *MBD5* causes diverse phenotypes which include hyperphagia, behavioural problems, craniofacial abnormalities, language impairment, microcephaly, development and motor delay, short stature, sleep disturbance, epilepsy, hyperphagia, obesity, and seizures [17–26]. The severity and complexity of the phenotypic spectrum of *MBD5* gene disruption varies from that of the high-penetrance 2q23.1 microdeletion [17–19], and incomplete penetrance for *MBD5* mutations has been identified in several previous report [17, 19]. Missense variants in *MBD5* (which perhaps have reduced penetrance) are associated with risk of autism spectrum disorders and may also contribute to schizophrenia and depression [26]. Obesity and hyperphagia are considered one of the most frequent clinical features (>50%) of 2q23.1 deletion syndrome [18, 21]. In addition, in a recent study of individuals with early-onset obesity, three copy number variants affecting this gene were identified [27].

Prompted by these findings, two further instances of possible oligogenic obesity were identified by re-analysis of WES data from 91 unrelated adults with severe obesity from the PMMO cohort (with severe obesity, but without identified learning disability). We note that in these two subsequent cases, variants affecting the *SH2B1* and *POGZ* genes are again involved. Further analysis in larger clinical cohorts will be required to determine whether this is a stochastic effect, or reflects some aspect of the functional implication of mutations in these genes: i.e. that a “second hit” (either genetic or environmental) may be required for penetrance.

Consistent with this, there is evidence that *SH2B1* mutations have incomplete or variable penetrance, supporting a possible requirement for other genetic and/or environmental factors to manifest the disease [28]. Individuals with variants in *SH2B1* exhibit a wide range of phenotypes including obesity, insulin resistance, and neurodevelopmental problems, while variants in other related obesity genes result in a diverse phenotypic spectrum which includes obesity.

We suggest that defects in more than one gene may be acting in concert to produce the observed phenotype in the individuals presented here. Recently, several diseases have been reported to show oligogenic inheritance, such as cardiovascular disease, autism, and Bardet-Biedl syndrome [3, 5–7].

There remain a number of obstacles to the use of mutational analysis in clinical practice for obesity, the most pressing being assessment of the clinical implications of variants of unknown significance (VUS). Here, several of the variants presented have not been previously reported and/or investigated for their functional effects, so would formally be reported as VUS. A further limitation of these analyses is that, since the PMMO participants are unrelated adults, DNA was not available for family segregation analysis. In this report, we used CADD scores, coupled with low MAF values in a large database of exome sequence data (gnomAD v2.1.1), to filter variants for likely pathogenicity. While the two cases identified here were of European ancestry, for whom ancestry-matched gnomAD allele frequencies were available, this approach currently has potential limitations for patients of non-European ancestry where less data on “normal” genetic variation is available. The genetics community awaits the development of methodologies for high throughput functional analyses of VUS to allow full interpretation of results, as well as large-scale analysis of sequenced and carefully phenotyped individuals of different ancestries [8]. Given the very low population-specific MAFs of the variants detected, however, it seems unlikely that these combinations are simply the result of chance. Additionally, further work will be required to ascertain whether some genes are more frequently involved in oligogenic obesity than other genes or whether this is a chance finding.

In summary, we present results suggesting that some cases of extreme obesity are due to a combination of mutations in more than one gene. This phenomenon could easily be missed in analysing data from candidate genes where it might be tempting to rely on a single explanatory mutation of known effect, without considering other (possibly heterozygous) VUS. As a result of our experience, we urge caution in interpretation of sequencing results from individual candidate genes which, in the instance of the family of proband 1, could have given rise to inaccurate genetic counselling. Utilisation of new tools that are being developed to explore the possibility of oligogenic inheritance may uncover additional examples in other conditions [29, 30]. WES or whole genome sequencing (WGS) allows exploration of a wider range of genes, and allows re-analysis as new causative genes are discovered, and where resources allow, should be the approach of choice.

## Materials and Methods

### Study participants and clinical information

#### Proband 1 and parents

A 22-year-old male proband of Pakistani heritage and his parents were seen at the Imperial College London Diabetes Centre in Abu Dhabi (Research Ethics Committee (IREC029)), for diagnostic purposes. Blood samples were collected from the patient and his parents. The proband has history of progressive weight gain starting in his mid-teens. He started to have problems with school performance at around 15 years of age. There was no family history of note and the parents were not consanguinous. On examination at age 10, he was noted to have generalised obesity (BMI 38.6 kg/m^2^) and prominent acanthosis nigricans. He had normal blood pressure and no Cushingoid features. Biochemical and hormonal investigations were normal, apart from profound hyperinsulinaemia. The patient was commenced on metformin with no response in terms of his weight. He was also seen by psychiatrists and was treated with various drugs, including antidepressants. The proband and his parents were enrolled in a clinical research study at the Imperial College London Diabetes Centre (ICLDC) in Abu Dhabi. WES was performed for the proband, followed by Sanger sequencing of the proband and his parents to confirm the presence of the variant and to check the segregation of the identified variant within the family.

#### PMMO cohort

The PMMO cohort (https://classic.clinicaltrials.gov/ct2/show/NCT01365416) is an observational research study of individuals with severe obesity (BMI > 40, or BM > 35 with at least one co-morbidity) who were ascertained from family doctors and/or undergoing bariatric surgery in a UK hospital-based service. The research has ethical approval from the NRES Committee London Riverside (Reference: 11/LO/0935/principal investigator: Professor Alexandra Blakemore) and informed consent was obtained from each subject [9]. A sub-group of 91 PMMO participants with BMI > 50 and no known syndromic features, were selected for a pilot WES study.

### Whole-exome sequencing and variant annotation

WES was performed using DNA from whole blood samples by the Genomics Service, MRC Clinical Sciences Centre, Imperial College London, UK. For this, an enriched library was prepared using SureSelectXT Human All Exon V4+UTRs and sequencing was run on a HiSeq25000 platform, generating 100 bp paired-end reads. FastQC version 0.10.0. was used to assess the quality of sequencing. Sequencing reads were mapped to the hg19 (GRCh37) reference genome through using BWA mem version 0.7.2. To refine the alignment Picard software (version 1.85) was used to remove duplicate reads and reduce false positive using BWA. For recalibration, realignments and variants calling the Genome Analysis Toolkit (GATK) were used. The annotated file was created using ANNOVAR.

For variant identification and analysis, variants were filtered and prioritised based on standard filtration and evaluation steps for WES data analysis as described previously in Alsters et al. [31]. This includes absence or low minor allele frequency in a public database (MAF < 1%), risk prediction by at least two out of four in silico prediction programs (Combined Annotation Dependent Depletion (CADD), Polyphen, Sorting Intolerant from Tolerant (SIFT) and, Proavean), and matching the relevant mode of inheritance for each gene [31, 32]. Subsequently, variants were re-screened against a list of monogenic obesity and syndromic obesity genes (see Supplementary Table 1). Sanger sequencing was carried out to confirm the presence of variants identified by WES, and to check for familial segregation in the parents of proband 1.

### Supplementary information

Supplementary table 1

## Data Availability

The datasets generated during and/or analysed during the current study are available from the corresponding author on reasonable request.
